# Electroencephalogram biomarkers as predictors of mortality and functional recovery in patients with severe traumatic brain injury: Protocol study

**DOI:** 10.1016/j.mex.2024.103146

**Published:** 2024-12-28

**Authors:** Walter Gomes da Silva Filho, Layza Julhia do Nascimento Moura, Arthur Barcelos Massariol Nascimento, Gabrielle Cristina Tessmann, Fabricia Silva Miranda, Vitória Caroline Reinoso de Almeida, Bárbara Vargens Broedel, Miller Lucas de Faria, Fernando Zanela da Silva Arêas

**Affiliations:** a Neurorehabilitation and Neuromodulation Laboratory, Department of Physiological Sciences, Federal University of Espírito Santo, City of Vitória, ES, Brazil; b Postgraduate program in Physiological Sciences, Federal University of Espírito Santo, City of Vitória, ES, Brazil; c Baylor Scott and White Research Institute and Baylor Scott and White Institute for Rehabilitation, Dallas, TX, USA

**Keywords:** Traumatic brain injury, Electroencephalogram, EEG biomarkers, Functional recovery, Clinical neuroscience, Data Colect Method

## Abstract

Traumatic brain injury (TBI) is a global public health condition that causes cognitive and behavioral deficits. This protocol assesses the potential of quantitative electroencephalogram (EEG) biomarkers, associated with inflammatory indicators, to predict mortality and functional recovery in patients with severe TBI. Through continuous monitoring and analysis of abnormal brain activity patterns, the protocol aims to personalize therapeutic interventions and improve patient quality of life. This randomized clinical trial includes 84 adult participants with severe TBI, followed at different stages of recovery, using validated scales for functional and predictive analysis. Traumatic brain injury (TBI) is a globally impactful public health condition characterized by initial brain injuries caused by traumatic forces, leading to cognitive and behavioral deficits. The trauma triggers inflammatory and neurochemical changes that exacerbate neuronal damage, resulting in neuropsychiatric complications. The use of electroencephalogram (EEG), particularly in its quantitative form (QEEG), is crucial for patients with severe TBI, as it allows early detection of abnormal brain activity patterns, such as slow waves, which indicate a worse prognosis. This continuous monitoring, combined with inflammatory biomarkers, guides personalized therapeutic interventions and improves the prediction of clinical outcomes, contributing to patient quality of life.

## Graphical abstract

**Figure:**
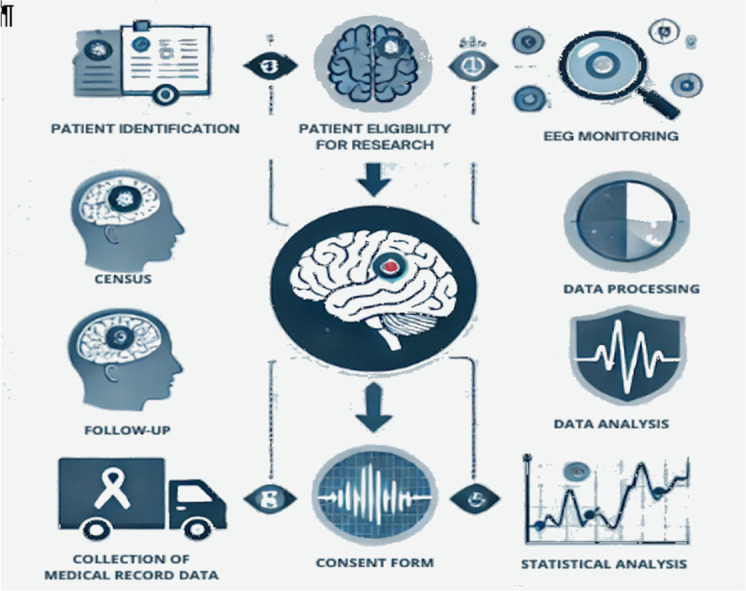

Specifications table

**Table:** 

Subject area:	Neuroscience
More specific subject area:	Traumatic Brain Injury
Name of your method:	Data Colect Method
Name of your protocol:	Inflammatory EEG Biomarkers in TBI
Reagents/tools:	TDCS/EEG HD Startsmis32
Experimental design:	This will be a randomized clinical trial of the prospective cohort type conducted in parallel with EEG monitoring.
Trial registration:	Informed consent was obtained from the participants.
Ethics:	Informed consent was obtained from all patients after patient eligibility was confirmed. The entire research project was submitted to the Research Ethics Committee of the Federal University of Espírito Santo, and approval was obtained in accordance with substantiated opinion number 4222,002.
Value of the Protocol:	The protocol aims to evaluate the predictive potential of EEG biomarkers in prognosis, outcomes, and functional recovery of patients with severe TBI, through comparison with a gold standard method.

## Background

Traumatic Brain Injury (TBI) remains a global health challenge, with high incidence rates in both developed and emerging economies [1,2]. Approximately 70 million people experience TBI annually, with 11% classified as severe [3,4]. In Brazil, between 2008 and 2019, the average annual incidence of TBI-related hospitalizations exceeded 131,000, with severe cases showing a hospital mortality rate of over 30%. TBI is the fifth leading cause of death among individuals aged 15–29 and the third among those aged 30–44, largely attributed to traffic accidents [5,6]. The Southeast region of Brazil reported the highest absolute incidence, with 648,447 cases during this period [7]. Effective clinical decision-making for moderate-to-severe TBI patients in intensive care units (ICUs) remains a significant challenge [8,9].

TBI is a complex condition that triggers substantial physiological changes, primarily an exaggerated inflammatory response. This inflammation often leads to secondary brain injuries, exacerbating neurological and functional complications [10,11]. Early Electroencephalography (EEG) monitoring at ICU admission is increasingly recognized as a valuable tool for assessing prognosis in severe TBI patients [8,9]. EEG provides real-time insights into brain activity, identifying specific patterns associated with injury severity and long-term outcomes [[12], [13], [14]].

EEG offers detailed information on cerebral function, including the detection of abnormal patterns such as diffuse slow waves and epileptiform activity. These abnormalities are linked to unfavorable outcomes, including cognitive deficits and a higher risk of post-traumatic epilepsy [[15], [16], [17]]. Additionally, EEG enables spatial analysis of electrical brain activity, highlighting asymmetries or regions of inactivity that correlate with neurological prognosis [18,19].

Despite the emphasis on acute-phase EEG monitoring, the subacute phase—post-ICU discharge—remains underexplored. Current studies provide a foundational understanding of EEG's relevance but lack focus on its application during recovery phases, where it may provide critical insights into functional recovery [20,21]. Addressing this gap is essential to advancing diagnostic accuracy and guiding therapeutic interventions for TBI patients.

This protocol seeks to evaluate the predictive potential of EEG biomarkers for prognosis, clinical outcomes, and functional recovery in severe TBI patients. By comparing EEG findings with gold-standard outcomes, this study aims to enhance understanding of the role of EEG in managing TBI and contribute to improved clinical care and recovery strategies.

## Description of protocol

This study aims to contribute to the understanding of the incidence of TBI and its associated biomarkers in a trauma care setting, with a particular focus on electroencephalography (EEG) as a potential diagnostic tool for early detection and monitoring of TBI. By integrating EEG data, the study seeks to provide valuable insights into the relationship between brain electrical activity and TBI, ultimately advancing clinical outcomes in the management of TBI patients.

This prospective cohort study will be conducted at a trauma reference hospital in Vitória, Espírito Santo, Brazil. The study population will include all patients admitted with traumatic injuries, who will be monitored by researchers through daily entries in their medical records. This will enable the identification of cases of traumatic brain injury (TBI) and determine the eligibility of patients for inclusion in the study. The inclusion and exclusion criteria are detailed in Table 1. Informed consent and comprehensive details about the study protocol will be provided to participants at the time of the first data collection, ensuring full understanding of the research procedures and objectives.

**Table 1:** Inclusion and exclusion criteria for the study.

Inclusion criteria	Exclusion criteria
Adults of both sexes, aged 18 or older	Under 18 years old
Glasgow Coma Scale (GCS) ≤ 8 at any time during hospital stay	Open TBI
Admitted to an Intensive Care Unit (ICU)	History of previous neurological diseases
Informed consent signed	History of previous psychiatric diseases

Sample size estimation was based on a two-tailed hypothesis test with a medium Pearson correlation coefficient (r = 0.3), a significance level (α) of 0.05, and a statistical power (1 - β) of 0.80. Using these parameters, a target sample size of 84 individuals was determined to provide adequate power for detecting clinically meaningful relationships between variables. The sample size calculation was performed using GPower software [22], a widely used tool for power analysis in experimental design.

## Data collection protocol

The EEG data collection protocol will involve monitoring patients with severe traumatic brain injury (TBI) during the subacute phase of the trauma. The subacute phase is defined as beginning on the seventh day following the traumatic event [[23], [24], [25]]. EEG recordings will be performed immediately after the patient's transfer from the intensive care unit (ICU) to the hospital ward. This timing was chosen to account for the complex and dynamic nature of the ICU environment, while the hospital ward is expected to offer a more stable and controlled setting for optimal EEG acquisition. The subacute phase was selected because, in comparison to the acute phase, patients are generally more stable during this period, which is also when key physiological recovery processes and neuronal reorganization typically occur [[26], [27], [28]].

To comprehensively assess the functional and cognitive status of patients throughout the study, functional scales will serve as a critical component for comparing and validating the predictive potential of EEG in relation to TBI events. These functional scales, as detailed in Table 2, will be administered at key time points: ICU discharge, hospital discharge, and at three, six, and twelve months post-TBI.

**Table 2:** Description of functional scales.

Scale	Description
Glasgow Coma Scale (GCS)	Assesses the patient's level of consciousness based on three parameters: eye opening, verbal response, and motor response. The score ranges from 3 to 15, with lower scores indicating more severe coma.
Dementia Rating Scale (DRS)	Used to assess cognitive function in dementia patients. The score is determined by the patient's performance on memory, orientation, and ability to perform daily activities. The score ranges from 0 to 144, with lower scores indicating greater severity.
Functional Independence Measure (MIF)	Assesses the functional independence level of patients in daily activities such as eating, dressing, and mobility. Scores range from 18 to 126, where 18 indicates total dependence and 126 indicates total independence.
Functional Ambulation Categories (FAC)	Measures the patient's ability to walk based on the support needed. Categories range from 0 (no walking) to 5 (independent walking).
Rancho Los Amigos Scale (RLA)	Assesses the level of neuropsychological recovery after a brain injury. There are seven levels ranging from total absence of response (Level I) to full recovery and return to social and professional activities (Level VII).

### EEG protocol

**Fig. 1:**
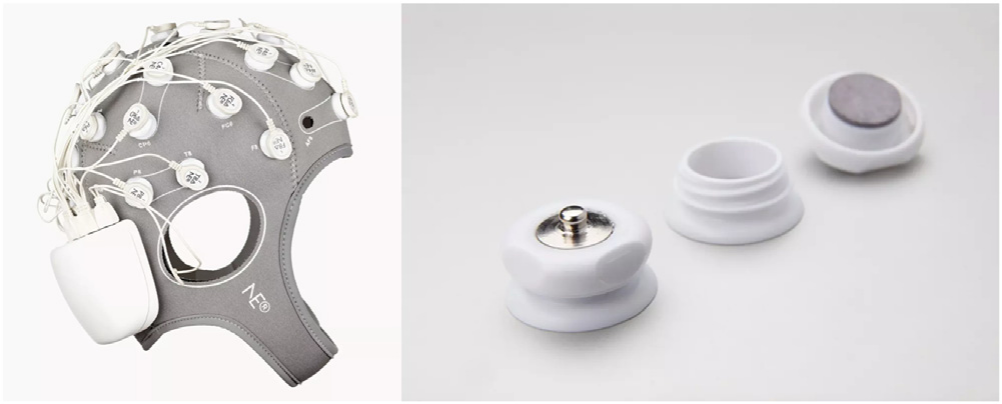
EEG equipment.

***Source:*** Neuroeletrics website

The EEG protocol will be performed with the patient in a resting, relaxed, and awake state, with eyes closed. EEG recordings will be conducted using the Startism 20 system (Starstim® tES-EEG systems), a 20-channel device with specialized electrodes designed for EEG monitoring (Fig. 1). The settings will include a time constant of 0.3 seconds and a high-frequency cutoff at 30 Hz. To minimize potential artifacts that could compromise the quality of the recordings, the EEG will be performed in a quiet environment with low lighting to ensure optimal conditions.

**Fig. 2:**
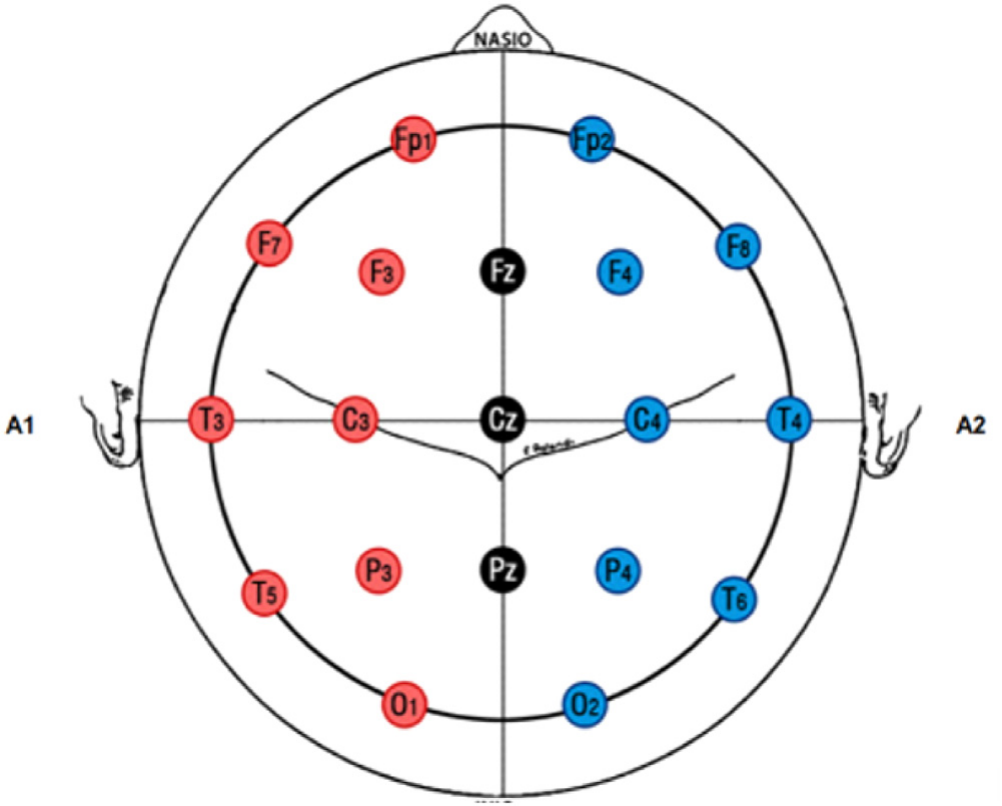
EEG montage according to the 10–20 system.

All cerebral electrical activity will be analyzed using computerized spectral EEG analysis and topographic brain mapping. Electrodes will be positioned according to the international 10–20 system, as shown in Fig. 2. The electrode impedance will be kept below 3 kΩ, with the earlobes serving as the reference. Prior to electrode placement, patients will be instructed to wash their hair with neutral shampoo and ensure it is dry, which will help minimize scalp impedance. During the electrode setup, conductive gel will be applied to the electrode sites to enhance signal quality and ensure proper contact with the scalp.

### EEG data recording and treatment

EEG data will be continuously recorded in a digital spectral file using the NIC2 software (Starstim® tES-EEG systems) and stored for subsequent analysis. The vigilance state of participants will be continuously monitored throughout the EEG recording process. The entire procedure will last 20 minutes, and all steps will be performed by a researcher trained in EEG techniques.

Biomarkers related to brain activity patterns will be considered for analysis in this study. Abnormal brain activity patterns will be evaluated for each participant and categorized, focusing on significant changes in the frequency and amplitude of brain waves across all monitored regions. Table 3 provides a detailed description of EEG wave patterns and their characteristics in relation to the monitored brain areas, functions, and potential alterations associated with traumatic brain injury (TBI).

**Table 3:** waveforms and functions in TBI.

Wave Type	Frequency (Hz)	Brain Region	Function	Alterations	Condition/Events	Interpretation	Waveform Example	References
Delta	0.5 - 4	Deep brain	Deep sleep, regeneration	Significant increase	Moderate to severe TBI	Indicates reduced cortical activity, coma state; impairment of brain function		Zetterberg, H., et al. (2013). *Nature Reviews Neurology*
Theta	4 -8	Hippocampus, temporal cortex	Creativity, meditation, light sleep	Increase	Mild to moderate TBI, concussion	Reflects altered consciousness and difficulties in attention/memory		Guskiewicz, K. M., et al. (2017). *Sports Medicine*
Alpha	08 – 12	Occipital cortex	Relaxation, alert state	Decrease	TBI and PTSD	Reduction is associated with difficulties in relaxation and hyperexcitability		Moffat, S. D., & Hampson, E. (2018). *Psychological Science*
Beta	12 - 30	Frontal, parietal cortex	Concentration, mental activity	Increase or irregularity	TBI with psychiatric sequelae	High levels indicate anxiety and cognitive overload; responses to post-traumatic stress		Kuperman, V., et al. (2015). *Neuropsychology*
Gamma	30 - 100	Various regions	Information processing	Irregular patterns	TBI with cognitive complications	Irregular patterns associated with processing disorders		Siegel, M., et al. (2012). *Nature Reviews Neuroscience*

### Collection and clinical information

Sociodemographic data for all participants will be collected from medical records, including information on age, gender, education level, occupation, and other relevant factors. Additionally, clinical data obtained during hospitalization will be recorded, such as vital signs, pupillary response characteristics, need for intubation, surgical interventions performed, imaging results, length of stay in the intensive care unit (ICU), and total hospitalization duration, among other clinical parameters. These data are crucial for a comprehensive characterization of the patients' social and clinical profiles. This detailed information will enable a more thorough analysis of the variables associated with traumatic brain injury (TBI) and will contribute to the development of more precise and individualized management strategies.

### Statistical analysis

The collected EEG data will be pre-processed using MATLAB software, equipped with the EEGLAB plugin (Neuroelectrics), for initial processing, including noise filtering, artifact removal, and extraction of specific features such as frequency and amplitude of brainwave bands [29]. Statistical analysis will involve the development of a predictive model based on categorized characteristics, such as frequency bands and amplitudes of EEG signals, to correlate these parameters with clinical outcomes [30].

Binomial logistic regression analyses will be performed using Stata (version 17) to explore the predictive power of EEG biomarkers. SPSS (version 28) will also be employed to conduct initial descriptive analyses, including variable distribution, normality tests (e.g., Shapiro-Wilk), and group comparisons using appropriate statistical tests (e.g., *t*-test, Mann-Whitney, or ANOVA, depending on data distribution). These analyses will ensure data adequacy and identify preliminary relationships between EEG parameters and clinical outcomes [31,32].

Additionally, ROC curves will be generated using SPSS, and the area under the curve (AUC) will be calculated to evaluate the sensitivity and specificity of predictive models. Model robustness will be validated through cross-validation, and additional tests, such as the Hosmer-Lemeshow test, will be applied to assess the logistic model's fit to the data [33].

### Protocol validation

The investigation of biomarkers associated with abnormal brain activity patterns in patients with severe traumatic brain injury (TBI) holds significant potential for both clinical practice and scientific research. Early identification of these patterns enables the anticipation of adverse clinical outcomes, improves prognostic assessment, and supports the development of more effective therapeutic strategies. This protocol provides a reliable methodological framework for future studies, with the potential to drive evidence-based interventions, improve clinical outcomes, and mitigate the public health burden of severe TBI.

## Limitations

The main limitation of this study is the lack of complementary materials with adequate methodological quality regarding the EEG biomarkers, which could provide more robust evidence to strengthen this protocol. In addition, the absence of consensus on which EEG biomarkers are most indicative of functional outcomes in patients with severe traumatic brain injury (TBI) remains a challenge for standardizing the analysis. The scarcity of previous studies in specific TBI populations, with varying etiologies and clinical characteristics, limits the generalization of findings to a broader context.

Another significant limitation is the difficulty in controlling all variables that may influence EEG activity, such as different treatments administered to patients, medication use, or even the presence of comorbidities. The variability in treatment protocols can influence EEG measurements, making the interpretation of results more complex and comparison with other studies more challenging.

Despite these limitations, the application of this protocol is both necessary and valid, as the clinical hypothesis, supported by indirect evidence, suggests that EEG biomarkers may be effective in predicting significant clinical outcomes in patients with severe TBI, with the potential to contribute to the development of new diagnostic and intervention strategies.

## Credit author statement

**Walter Gomes da Silva Filho:** Conceptualization, Methodology, and Original Draft Preparation; **Layza Julhia do Nascimento Moura:** Data Collection, Patient Monitoring, and Manuscript Revision; **Arthur Barcelos Massariol Nascimento:** Writing and Editing; **Gabrielle Cristina Tessmann:** Data Collection and Patient Monitoring; **Fabricia Silva Miranda:** Writing and Manuscript Revision; **Vitória Caroline Reinoso de Almeida:** Data Collection and Data Processing; **Isabela Loes Batista Maia:** Data Collection and Patient Monitoring; **Bárbara Vargens Broedel:** Data Collection and Patient Monitoring; **Miller Lucas de Faria:** Data Collection and Patient Monitoring; **Fernando Zanela da Silva Arêas:** Supervision, Manuscript Revision, and Editing.

## Declaration of competing interest

The authors declare that they have no known competing financial interests or personal relationships that could have appeared to influence the work reported in this paper.

## Data Availability

Data will be made available on request.
